# The Effect of Music Therapy on Postoperative Pain and Agitation During Septorhinoplasty: A Blinded Clinical Trial

**DOI:** 10.1002/hsr2.70716

**Published:** 2025-04-21

**Authors:** Sevil Nasirmohtaram, Maryam Akbari, Mir Mohammad Jalali, Arman Parvizi

**Affiliations:** ^1^ Department of Otolaryngology and Head and Neck Surgery, School of Medicine, Otorhinolaryngology Research Center Amiralmomenin Hospital, Guilan University of Medical Sciences Rasht Iran; ^2^ Department of Anesthesiology, Anesthesiology Research Center, Alzahra Hospital Guilan University of Medical Sciences Rasht Iran

**Keywords:** agitation, music therapy, pain, septorhinoplasty, surgery

## Abstract

**Background and Aims:**

It has been shown that patients who need surgery, such as septorhinoplasty candidates, suffer from unbearable pain during and after surgery. One of the main considerations of the surgeons for these patients is making them experience a pleasant, comfortable, and painless intervention during septorhinoplasty utilizing nonpharmacological or pharmacological approaches. This study aimed to investigate the antianxiety and analgesic effects of listening to music as a nonpharmacological method in patients undergoing septorhinoplasty with general anesthesia.

**Methods:**

In this randomized controlled clinical trial study, 80 patients who were candidates for septorhinoplasty were randomly categorized into the case (with music) and control groups (without music) from May to September 2022. STATA Version 14.0 (StataCorp) was used for the statistical analysis of the acquired data. Pain perception and agitation were evaluated using the Visual Analog Scale (VAS) and Sedation‐Agitation Scale (SAS), respectively, and compared between the two groups during the early postoperative period.

**Results:**

Although the baseline SAS score in the two groups was similar, findings showed a significantly lower score in the intervention group in recovery units (*t*‐test 4.30, *p* < 0.001). The mean VAS was 0.8 ± 1.18 and 1.87 ± 1.13 in the intervention and control groups, respectively (*p* < 0.001). The satisfaction level of those patients in the intervention group was demonstrated during the procedure of the intervention. However, there was no significant difference between the control and intervention groups for hemodynamic parameters.

**Conclusion:**

Music therapy during surgical procedures can postoperatively simplify the optimized postsurgical outcomes for patients undergoing septorhinoplasty.

**Trial Registration:** Iranian Registry of Clinical Trials (IRCT20210307050609N2)

## 1 Introduction

In aesthetic surgeries, for example, septorhinoplasty, compared to other elective surgeries, experiencing a pleasant intervention and reducing pain is much more concern, which leads surgeons toward nonpharmacological approaches in addition to medications, including complementary and alternative medicine aimed to conduct a comfortable intervention [1, 2]. In addition to pain, anxiety, which is a physiological response in the preoperative period and a factor associated with pain, also affects the level of patient satisfaction and should not be ignored.

It is noteworthy that anxiety causes tachycardia, tachypnea, and hypertension, as well as an increased catecholamine release during the surgery day. It has been interrogated that clinical usage of some medications aimed at the reduction of postoperative pain and preoperative stress is not of sufficient clinical efficacy, pushing surgeons to benefit from alternative medicine [3, 4].

To achieve more effective methods, complementary medicine opened a promising window to music therapy, which has been used in clinical practice as a nonpharmacological and adjuvant approach, either as a complementary/alternative therapy (naturopathic treatment) in clinical practices or as opioid‐free anesthesia (especially for older or critically ill patients), and approved by American Music Therapy Association [4, 5, 6, 7, 8, 9, 10, 11]. Although patients are typically amnestic during general anesthesia, results of a wide array of research corroborated that auditory stimuli like music therapy can be sensed and neurologically processed during general anesthesia independent of the surgery type. This finding may be responsible for either memory formation while unaware or the amount of analgesic consumption [1, 12, 13]. Interestingly, the beneficial effects of music have been recorded in a wide array of studies, resulting in improved postoperative recovery, lowered psychological stress, and reduced analgesic consumption [1, 3, 13, 14, 15, 16, 17]. Also, music has been historically utilized in the Olympic Games as a motivator to enhance physical potential. In the medical era, it may be applied as an inexpensive, safe, and noninvasive intervention for reducing pain, anxiety, or agitation in patients who undergo a surgical procedure [18, 19].

Altogether, according to the aforementioned points, the present study aimed to evaluate the anxiolytic and analgesic effects of listening to music during general anesthesia in patients who are clinically diagnosed one with septorhinoplasty surgery, and assess the changes in hemodynamic parameters of those patients.

## 2 Methods

### 2.1 Study Design and Sample

This study was designed as a blinded RCT and an experimental study to evaluate the anxiolytic and analgesic effects of listening to music during general anesthesia in patients with septorhinoplasty surgery. This study hypothesizes that music during surgery will reduce the pain and agitation of patients after septorhinoplasty. This study was approved by the Ethics Committee of Guilan University of Medical Sciences (no.IR.GUMS.REC.1400.538). Also, the trial was registered at the Iranian Registry of Clinical Trials (IRCT20210307050609N2). Then, an oral conscience and written informed consent (signed forms) were achieved. They were ensured about the usage of the collected data (confidentiality of their personal information) solely for the study, and their human right to withdraw from this study at any time.

The sample size for this study was determined using the G*power software (version 3.1.9.2,) program with a 5% margin of error and *β* of 0.02. According to Gökçek's research [14] and considering the possibility of 10% dropout, the sample size was estimated to be 80 patients (40 patients in each group).

**Figure 1:**
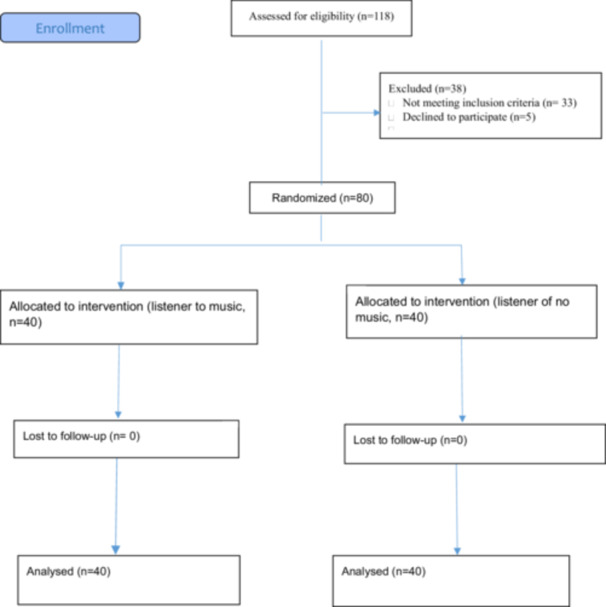
Study flowchart.

Figure 1 demonstrates the flow diagram of patients through the trial according to the consolidated standards of reporting trial statements.

### 2.2 Participants

Eighty patients who were in the age range of 18–65 years old had been clinically diagnosed for undergoing a septorhinoplasty surgery (diagnostic criteria according to the ones outlined and clarified by the Helsinki Declaration) and admitted to clinics of our university‐affiliated hospital from May to September 2022 were included in this study. The ones who had been clinically diagnosed with or had been suffering from hearing disorders/cognitive disorders/psychological disorders, those who had been paraclinically diagnosed with alcohol abuse or a history of addiction, as well as the ones who had received antidepressant or anxiolytic agents, were excluded from this study. Moreover, the ones who had been clinically diagnosed with predisposition disorders like any type of cancer were excluded. Also, those patients who had required complex techniques, for example, rib cartilage harvesting, were not included in this study.

### 2.3 Randomization and Blinding

After obtaining oral and written informed consent, a checklist of demographic data was fulfilled at the admission of the patients to the operation ward. Block randomization was used to divide the patients using PASS software. Randomization was performed by a researcher who was not involved in the clinical stages of the project. The patients were randomized 1:1 to the groups either listening to the music (case or intervention group) or control group (listening to nothing), with block randomization in blocks of four. The randomization and blinding were carried out by an independent assistant. Researchers were able to be blinded in this study due to their inactive involvement in sampling and collecting the studied participants for this study, implementation of interventions, and data collection procedures. All patients were operated under the supervision of a single surgeon and an open approach, and the clinically used anesthesia technique was the same, being conducted by the same anesthesiologist.

### 2.4 Intervention

All patients were operated under general anesthesia with total intravenous anesthesia (TIVA—including midazolam 1 mg, propofol 2.5 mg/kg, lidocaine 20–40 mg, and cis‐atracurium 0.1–0.2 mg/kg). Anesthesia maintenance was retained by remifentanil 0.1 μg/kg/min and propofol 50–150 μg/kg/min until mean arterial pressure (MAP) was retained in a range of 85–100 mmHg. Patients were positioned in a reverse Trendelenburg position. A solution of epinephrine 1/100,000 and lidocaine 1% were infiltrated in the location of marginal incisions and nasal septum before consuming surgery, and no additional vasoconstrictor was used. All of the patients (in both ears) and the surgeon (just in one ear) were equipped with airpods technically promoted to the technology of noise canceling system.

In the music group, Apple AirPods were placed in both ears of the patient and each ear was presented with identical music from a selected playlist of classical piano music (with an average rhythm of 80 beats/min) from the player (Apple mobile). The music volume was 65 dB.

For the control group, only silence was played. Among the options in Iranian music styles, which include: classical (traditional, nontraditional), rock, hip‐pop, or rap and pop, classical music was selected based on the research team's preference for nonverbal music (given the possibility of the impact of the verbal content of the song being played), and in all patients, nontraditional classical music was chosen.

### 2.5 Outcome Measurement

Anxiety and pain during surgery and intubation were measured based on the assessment of changes in the patient's hemodynamic parameters. The hemodynamic parameters were clinically measured every 15 min. The surgery and the total anesthesia time were recorded for each patient in both groups. The Sedation‐Agitation Scale (SAS) was assessed at the extubation time, and also 5 min after entrance of the patients to our recovery ward. Additionally, pain, based on the Visual Analog Scale (VAS), and general satisfaction of patients from surgery were recorded in our recovery unit.

### 2.6 Data Analysis

STATA Version 14.0 (StataCorp) was used for the statistical analysis. A *t*‐test was used to assess independent quantitative determinants in each group. Categorical variables (nonparametric determinants) were expressed with mean ± standard deviation (SD) and percentage (%). Results were expressed as mean ± SD. Statistical significance was considered at *p* < 0.05.

## 3 Results

After screening for exclusion criteria, 80 patients were randomized into 2 groups (including 40 patients as listeners to the music (case or intervention group) and 40 patients as listeners to nothing (control group). In this study, there were no dropouts and no lost to follow‐up. Table 1 summarizes the demographic data of the studied population in this study at the baseline. There were no statistically significant differences between the control and intervention groups in terms of baseline parameters. The mean SAS during extubation time was 3.22 ± 0.97 and 3.62 ± 1.14 for the intervention and control groups, respectively. Furthermore, there were no significant differences between the control and intervention groups for SAS at the first time point (extubation time) (*p* = 0.09) However, the mean SAS in the recovery ward was 2.07 ± 0.91 and 2.95 ± 0.90 for the intervention and control groups, respectively, [at the first time point (95% CI: 2.91–3.53), and at the second time point (95% CI: −1.28 to 0.46)]. Moreover, there was a significant difference between the control and intervention groups for lower SAS at the second time point (in the recovery ward) (*p* < 0.001). The mean VAS during extubation time was 0.8 ± 1.18 and 1.87 ± 1.13 for the intervention and control groups, respectively. Additionally, there was a significant difference between the control and intervention groups for VAS at the first time point (extubation time) (*p* < 0.001).

**Table 1:** Baseline demographics.

	Music group	Control	All patients	*p*
Age, year	29.87 ± 7.88	31.45 ± 9.28	30.66 ± 8.59	0.41
Female, *n* (%)	38	36	74	0.72
MAP0^a^	70.4 ± 13.27	74.8 ± 14.03	72.6 ± 13.75	0.15
HR0^b^	84.15 ± 9.86	81.12 ± 12.72	82.63 ± 10.45	0.19

a Mean of mean arterial pressure at baseline.

b Mean heart rate at baseline.

Overall satisfaction at the end of surgery in recovery unit after full consciousness was assessed by asking the patient a question with a choice between poor, good, and excellent. The level of patient satisfaction was 87.5% for the whole procedure of surgery process in the case group compared to the control group (57.5%). There were no significant differences between the control and intervention groups for the mean arterial blood pressure and heart rate during the surgery. There were no significant differences between the control and intervention groups for the mean total time of the surgery (59.75 ± 12.08 min and 64.47 ± 12.96 min, respectively) (*p* = 0.09) (Table 2).

**Table 2:** Comparisons of various outcomes among music (*n* = 38) and control (*n *= 36) groups.

	Music group, mean (SD)	Control group, mean (SD)	Difference, mean (95% CI)	*p*
SAS_extub_	3.23 (0.97)	3.62 (1.14)	−0.40 (−0.87; 0.07)	0.09
SAS_recovery_	2.07 (0.91)	2.95 (0.90)	−0.88 (−1.28; −0.47)	< 0.001
VAS	0.80 (1.18)	1.87 (1.13)	−1.08 (−1.59; −0.56)	< 0.001
Mean total time	64.47 (12.96)	59.75 (12.08)	4.73 (−0.86; 10.31)	0.09

Abbreviations: SAS_extub_, sedation‐agitation score (SAS) during extubating; SAS_recovery_, sedation‐agitation score (SAS) in recovery ward; VAS, pain based on Visual Analog Scale.

## 4 Discussion

Aesthetic surgeons always sought methods aimed at amelioration of the preoperative stressful situation and reduction of postoperative pain after cosmetic surgeries. On the one hand, septorhinoplasty is not an exception. On the other hand, an exaggerated or prolonged response to stress or unwanted acute pain can lead to detrimental effects on metabolic and hormone states, which is harmful to cardiovascular and immune systems and a life‐threatening factor for patients who underwent a surgical procedure [20]. In a clinical study, Choi et al. [21] tried to demonstrate the effects of classical music therapy on the quality of recovery and clinical management of postoperative pain among patients who had undergone gynecological laparoscopy under spinal anesthesia. They investigated five criteria (including emotions, pain, physical comfort, support, and independence) 1 day postoperatively on 41 patients as a case group. Moreover, they investigated pain, nausea, and vomiting 30 min, 3 h, 1 and 3 days postoperatively. They reported a lower score in the pain criteria among the case group, especially 3 days postoperatively [21]. The results of this study generally corroborated our results in the case of recovery of postoperative pain after septorhinoplasty. Similarly, results of a controlled, randomized study conducted by Hepp et al. [22] investigating the effects of interventional therapies like music during cesarean delivery on several criteria like anxiety and stress of the mothers, demonstrated lower anxiety at skin suture and even 2 h after surgery in the case group (listeners to the music) regarding SAS and VAS scales [22] generally corroborating our results in case of recovery of postoperative pain after septorhinoplasty. The same interpretation goes back to the results acquired from a study conducted by Isni Maftuhah and Saryono [23], who tried to investigate the effects of interventional therapies like music aimed at the reduction of postoperative pain and depression among patients suffering from benign prostate hyperplasia. The results of this study generally corroborated our results in the recovery of postoperative pain after septorhinoplasty.

Although music therapy (either in the form of receptive or group therapy, even the patient‐preferred live ones) has been proposed in some studies as an effective intervention to improve postoperative patient conditions, quality of sleep, and enhanced recovery after emergency surgeries or after periodontal flap surgeries in the postanesthesia care unit (reanimation or recovery unit), evidence in this field is sparse and uncertain [18, 24, 25, 26, 27, 28, 29, 30, 31, 32, 33, 34, 35, 36]. In this prospective randomized clinical trial study, we evaluated the effect of music on pain sensation and agitation (SAS scale) in the early postoperative hour. To omit the confounding effects of the background noise of the anesthesia machine and conversations, a noise‐canceling system was activated. At the first time point, which was precisely during extubation, there was no significant effect of intervention observed on the score of SAS. This finding may be due to the more influential effects of anesthetic and analgesic medications used during and at the termination of the procedure. According to the anesthesia regimen each anesthesiologist selects, specific physiologic responses are expected. This finding was also represented in hemodynamic parameters in our patients. On the opposite side, as the effect of medications diminishes, the role of paramedical interventions becomes more noticeable. As reported in this study, during observation in the recovery ward, patients who were listening to music during surgery were more self‐possessed, achieved total consciousness in tranquility, and reported less feeling of pain. In this study, we could not find any correlation between total surgery time and the intervention group. This finding contradicts the results of the systematic review of EL Boghdady and Ewalds‐Kvist [37]. One explanation for this finding may be that the surgeries are performed by the same surgeon with her principles and unique set of techniques that have tiny modifications based on the patient's deformities. Additionally, patients demanding special techniques were ruled out at the beginning of the study.

### 4.1 Limitations

One of the limitations of the present study was the fact that the effect of medications used during the operation could not be completely ignored, as there may be some minor modifications in some instances based on the patient's conditions. Further studies are recommended to generalize the results of our study to other elective procedures such as septorhinoplasty. It may be feasible to assess patients a few days after surgery using valid questionnaires to evaluate the possible long‐term effects of the intervention on the psychosocial feedback of patients from surgery as a stressful event. To address the gaps in current strategies aimed at patient care and achieving optimal clinical outcomes for better management of pain and discomfort during septorhinoplasty surgeries, further research and interdisciplinary collaboration between specialists from different disciplines and health system coordinators are needed and strongly recommended.

## 5 Conclusion

The present study revealed that playing music during surgery results in a better postsurgical condition and a more favorable experience of the whole procedure for patients undergoing septorhinoplasty. It seems that our intervention was clinically influential in achieving better recovery and clinical outcomes of the anesthesia and a shorter period for the development of consciousness for the aforementioned patients.

## Author Contributions

**Sevil Nasirmohtaram:** conceptualization, data curation, supervision, project administration, writing – original draft, writing – review and editing. **Maryam Akbari:** investigation, data curation, writing – review and editing, writing – original draft, methodology, validation. **Mir Mohammad Jalali:** validation, formal analysis, methodology, supervision, project administration. **Arman Parvizi:** data curation, resources, writing – review and editing, writing – original draft. All authors have read and approved the final version of the manuscript.

## Ethics Statement

This study was conducted following the Declaration of Helsinki and was approved by the Ethics Committee of Guilan University of Medical Sciences (no.IR.GUMS.REC.1400.538).

## Conflicts of Interest

The authors declare no conflicts of interest.

## Transparency Statement

The lead author Maryam Akbari affirms that this manuscript is an honest, accurate, and transparent account of the study being reported; that no important aspects of the study have been omitted; and that any discrepancies from the study as planned (and, if relevant, registered) have been explained.

## Data Availability

The data sets used and/or analyzed during the current study are available from the corresponding author upon reasonable request.

## References

[1] V. X. Fu , P. Oomens , D. Sneiders , et al., “The Effect of Perioperative Music on the Stress Response to Surgery: A Meta‐Analysis,” Journal of Surgical Research 244 (2019): 444–455. 31326711 10.1016/j.jss.2019.06.052

[2] W. M. Sin and K. M. Chow , “Effect of Music Therapy on Postoperative Pain Management in Gynecological Patients: A Literature Review,” Pain Management Nursing 16, no. 6 (2015): 978–987. 26697822 10.1016/j.pmn.2015.06.008

[3] M. L. Diller and V. Master , “Integrative Surgery: Embedding Complementary and Nonpharmacologic Therapies Into Surgical Pain Management Strategies,” American Surgeon 89, no. 2 (2023): 192–196. 35816178 10.1177/00031348221110244

[4] Y. Liu and M. A. Petrini , “Effects of Music Therapy on Pain, Anxiety, and Vital Signs in Patients After Thoracic Surgery,” Complementary Therapies in Medicine 23, no. 5 (2015): 714–718. 26365452 10.1016/j.ctim.2015.08.002

[5] H. van der Wal‐Huisman , K. S. K. Dons , R. Smilde , E. Heineman , and B. L. van Leeuwen , “The Effect of Music on Postoperative Recovery in Older Patients: A Systematic Review,” Journal of Geriatric Oncology 9, no. 6 (2018): 550–559. 29678668 10.1016/j.jgo.2018.03.010

[6] S. H. Khan , M. Kitsis , D. Golovyan , et al., “Effects of Music Intervention on Inflammatory Markers in Critically Ill and Post‐Operative Patients: A Systematic Review of the Literature,” Heart & Lung 47, no. 5 (2018): 489–496. 30001799 10.1016/j.hrtlng.2018.05.015 PMC6380515

[7] F. Giordano , M. Giglio , I. Sorrentino , et al., “Effect of Preoperative Music Therapy Versus Intravenous Midazolam on Anxiety, Sedation and Stress in Stomatology Surgery: A Randomized Controlled Study,” Journal of Clinical Medicine 12, no. 9 (2023): 3215. 37176656 10.3390/jcm12093215 PMC10179016

[8] J. Y. Song , H. Choi , M. Chae , J. Ko , and Y. E. Moon , “The Effect of Opioid‐Free Anesthesia on the Quality of Recovery After Gynecological Laparoscopy: Study Protocol for a Prospective Randomized Controlled Trial,” Trials 22, no. 1 (2021): 1–8. 33712080 10.1186/s13063-021-05166-z PMC7953824

[9] G. Durust Sakalli and O. Kara , “Use of Complementary and Integrative Methods in the Management of Postoperative Pain: A Narrative Literature Review,” Mediterranean Nursing and Midwifery 2, no. 2 (2022): 84–93.

[10] A.‐K. Lederer , C. Schmucker , L. Kousoulas , S. Fichtner‐Feigl , and R. Huber , “Naturopathic Treatment and Complementary Medicine in Surgical Practice,” Deutsches Arzteblatt International 115, no. 49 (2018): 815–821. 30678751 10.3238/arztebl.2018.0815 PMC6369237

[11] N. Stoicea , T. J. Gan , N. Joseph , et al., “Alternative Therapies for the Prevention of Postoperative Nausea and Vomiting,” Frontiers in Medicine 2 (2015): 87. 26734609 10.3389/fmed.2015.00087 PMC4679858

[12] V. X. Fu , K. J. Sleurink , J. C. Janssen , B. P. L. Wijnhoven , J. Jeekel , and M. Klimek , “Perception of Auditory Stimuli During General Anesthesia and Its Effects on Patient Outcomes: A Systematic Review and Meta‐Analysis,” Canadian Journal of Anesthesia/Journal Canadien d'Anesthésie 68, no. 8 (2021): 1231–1253. 10.1007/s12630-021-02015-0 PMC8282577 34013463

[13] K. Sandhu and H. Dash , “Awareness During Anaesthesia,” Indian Journal of Anaesthesia 53, no. 2 (2009): 148–157. 20640115 PMC2900098

[14] E. Gökçek and A. Kaydu , “The Effects of Music Therapy in Patients Undergoing Septorhinoplasty Surgery Under General Anesthesia,” Brazilian Journal of Otorhinolaryngology 86 (2020): 419–426. 31523022 10.1016/j.bjorl.2019.01.008 PMC9422617

[15] A. Ortega , F. Gauna , D. Munoz , G. Oberreuter , H. A. Breinbauer , and L. Carrasco , “Music Therapy for Pain and Anxiety Management in Nasal Bone Fracture Reduction: Randomized Controlled Clinical Trial,” Otolaryngology–Head and Neck Surgery 161, no. 4 (2019): 613–619. 31184266 10.1177/0194599819856604

[16] H.‐J. Trappe , “The Effects of Music on the Cardiovascular System and Cardiovascular Health,” Heart 96, no. 23 (2010): 1868–1871. 21062776 10.1136/hrt.2010.209858

[17] C. K. Cheung , J. O. Adeola , S. S. Beutler , and R. D. Urman , “Postoperative Pain Management in Enhanced Recovery Pathways,” Journal of Pain Research 15 (2022): 123–135. 35058714 10.2147/JPR.S231774 PMC8765537

[18] J. Hole , M. Hirsch , E. Ball , and C. Meads , “Music as an Aid for Postoperative Recovery in Adults: A Systematic Review and Meta‐Analysis,” Lancet 386, no. 10004 (2015): 1659–1671. 26277246 10.1016/S0140-6736(15)60169-6

[19] K. Nelson , D. Bruene , M. Adamek , K. Kooi , and K. Hawkins , “Music Therapy and Post‐Operative Pain Management: Improved Outcomes,” Pain Management Nursing 22, no. 2 (2021): 235–236.

[20] K. Muawanah and S. Sulastri , “Controlling Post‐Operative Pain With Early Mobilization and Music Therapy,” Jurnal Kesehatan 14, no. 1 (2023): 150–156.

[21] E. K. Choi , J. Baek , D. Lee , and D. Kim , “Effect on Music Therapy on Quality of Recovery and Postoperative Pain After Gynecological Laparoscopy,” Medicine 102, no. 9 (2023): e33071. 36862891 10.1097/MD.0000000000033071 PMC9981401

[22] P. Hepp , C. Hagenbeck , J. Gilles , et al., “Effects of Music Intervention During Delivery on Anxiety and Stress of the Mother a Controlled, Randomised Study,” BMC Pregnancy and Childbirth 18 (2018): 1–8. 30390639 10.1186/s12884-018-2069-6 PMC6215648

[23] I. Maftuhah and S. Saryono , “Music Interventions to Reduce Pain in Postoperative Patients Benigna Prostate Hyperplasia,” British Journal of Nursing Studies 3, no. 1 (2023): 38–44.

[24] E. R. Gasenzer , A. Kanat , and E. Neugebauer , “Neurosurgery and Music; Effect of Wolfgang Amadeus Mozart,” World Neurosurgery 102 (2017): 313–319. 28242489 10.1016/j.wneu.2017.02.081

[25] M. Kahloul , S. Mhamdi , M. S. Nakhli , et al., “Effects of Music Therapy Under General Anesthesia in Patients Undergoing Abdominal Surgery,” Libyan Journal of Medicine 12, no. 1 (2017): 1260886. 28452603 10.1080/19932820.2017.1260886 PMC5328375

[26] D. M. Drzymalski , M. Dahlawi , R. R. Hall , S. Ranjan , and C. L. Best , “The Effect of Mozart Music on Patient Satisfaction During Caesarean Delivery: A Randomised Controlled Trial,” Anaesthesiology Intensive Therapy 55, no. 1 (2023): 114–119. 37409839 10.5114/ait.2023.129007 PMC10415609

[27] K. Schou , Music Therapy for Post Operative Cardiac Patients: A Randomized Controlled Trial Evaluating Guided Relaxation With Music and Music Listening on Anxiety, Pain, and Mood (InDiMedia, 2008).

[28] N. Nuriya , G. N. Alivian , A. Taufik , and S. Saryono , “Music Therapy to Reduce Pain Intensity in Post Fracture Surgery Patients: Systematic Review,” International Journal of Biomedical Nursing Review 1, no. 2 (2023): 101–124.

[29] K. Madan and R. B. Sriram , “Pain Management in Enhanced Recovery After Emergency Surgery,” In Topics in Postoperative Pain (IntechOpen, 2023).

[30] A. T. Masoud , A. Samy , A. M. Elshrery , E. Taher , K. H. Shaker , and A. M. Abbas , “The Effect of Music on Pain Perception in Women Scheduled for Elective Cesarean Section: A Systematic Review and Meta‐Analysis,” Proceedings in Obstetrics and Gynecology 10, no. 1 (2020): 1.

[31] J. Luo and S. Min , “Postoperative Pain Management in the Postanesthesia Care Unit: An Update,” Journal of Pain Research 10 (2017): 2687–2698. 29180895 10.2147/JPR.S142889 PMC5695271

[32] M. A. Schneider , “The Effect of Listening to Music on Postoperative Pain in Adult Orthopedic Patients,” Journal of Holistic Nursing 36, no. 1 (2018): 23–32. 29436975 10.1177/0898010116677383

[33] H. Tang , L. Chen , Y. Wang , Y. Zhang , N. Yang , and N. Yang , “The Efficacy of Music Therapy to Relieve Pain, Anxiety, and Promote Sleep Quality, in Patients With Small Cell Lung Cancer Receiving Platinum‐Based Chemotherapy,” Supportive Care in Cancer 29, no. 12 (2021): 7299–7306. 34041615 10.1007/s00520-021-06152-6

[34] F. Lagattolla , B. Zanchi , M. Pietro , et al., “Receptive Music Therapy Versus Group Music Therapy With Breast Cancer Patients Hospitalized for Surgery,” Supportive Care in Cancer 31, no. 3 (2023): 162. 36781543 10.1007/s00520-023-07624-7 PMC9924845

[35] P. Punnyamol , S. Ahamed , G. Sudhakaran , M. Shilpalakshmi , H. Ali , and A. Renji , “Effect of Music Therapy on Patients Undergoing Periodontal Flap Surgery,” Journal of Dr. NTR University of Health Sciences 11, no. 4 (2022): 281–287.

[36] M. Merry and M. J. Silverman , “Effects of Patient‐Preferred Live Music on Positive and Negative Affect and Pain With Adults on a Post‐Surgical Oncology Unit: A Randomized Study,” Arts in Psychotherapy 72 (2021): 101739.

[37] M. El Boghdady and B. M. Ewalds‐Kvist , “The Influence of Music on the Surgical Task Performance: A Systematic Review,” International Journal of Surgery 73 (2020): 101–112. 31760139 10.1016/j.ijsu.2019.11.012

